# Mass Spectrometry-Based Metabolomics of Phytocannabinoids from Non-Cannabis Plant Origins

**DOI:** 10.3390/molecules27103301

**Published:** 2022-05-20

**Authors:** Sarana Rose Sommano, Piyachat Sunanta, Noppol Leksawasdi, Kittisak Jantanasakulwong, Pornchai Rachtanapun, Phisit Seesuriyachan, Yuthana Phimolsiripol, Korawan Sringarm, Warintorn Ruksiriwanich, Pensak Jantrawut, Chuda Chittasupho

**Affiliations:** 1 Plant Bioactive Compound Laboratory, Faculty of Agriculture, Chiang Mai University, Chiang Mai 50100, Thailand; sunantapiya@gmail.com; 2 Cluster of Agro Bio-Circular-Green Industry (Agro BCG), Chiang Mai University, Chiang Mai 50100, Thailand; noppol@hotmail.com (N.L.); jantanasakulwong.k@gmail.com (K.J.); pornchai.r@cmu.ac.th (P.R.); phisit.s@cmu.ac.th (P.S.); yuthana.p@cmu.ac.th (Y.P.); korawan.s@cmu.ac.th (K.S.); 3 Cluster of Research and Development of Pharmaceutical and Natural Products Innovation for Human or Animal, Chiang Mai University, Chiang Mai 50200, Thailand; warintorn.ruksiri@cmu.ac.th (W.R.); pensak.j@cmu.ac.th (P.J.); chuda.c@cmu.ac.th (C.C.); 4 School of Agro-Industry, Faculty of Agro-Industry, Chiang Mai University, Chiang Mai 50100, Thailand; 5 Department of Animal and Aquatic Science, Faculty of Agriculture, Chiang Mai University, Chiang Mai 50100, Thailand; 6 Department of Pharmaceutical Sciences, Faculty of Pharmacy, Chiang Mai University, Chiang Mai 50200, Thailand

**Keywords:** *Cannabis sativa* L., cannabinomic, endocannabinoid, phytocannabinoids, mass spectrometry, metabolite profiling

## Abstract

Phytocannabinoids are isoprenylated resorcinyl polyketides produced mostly in glandular trichomes of *Cannabis sativa* L. These discoveries led to the identification of cannabinoid receptors, which modulate psychotropic and pharmacological reactions and are found primarily in the human central nervous system. As a result of the biogenetic process, aliphatic ketide phytocannabinoids are exclusively found in the cannabis species and have a limited natural distribution, whereas phenethyl-type phytocannabinoids are present in higher plants, liverworts, and fungi. The development of cannabinomics has uncovered evidence of new sources containing various phytocannabinoid derivatives. Phytocannabinoids have been isolated as artifacts from their carboxylated forms (pre-cannabinoids or acidic cannabinoids) from plant sources. In this review, the overview of the phytocannabinoid biosynthesis is presented. Different non-cannabis plant sources are described either from those belonging to the angiosperm species and bryophytes, together with their metabolomic structures. Lastly, we discuss the legal framework for the ingestion of these biological materials which currently receive the attention as a legal high.

## 1. Introduction

The recent advance in metabolomics including application devices of liquid or gas chromatography-coupled with mass spectrometry (LC- or GC-MS) or nuclear magnetic resonance spectroscopy (NMR) together with bioinformatic and chemometric approaches has prompted the understanding of the metabolite profiling of cannabis plants and related species [1,2,3]. More than 500 constituents have recently been elucidated in the cannabis species, including terpenes (mono-, di-, sesqui-and triterpenes), flavonoids, alkanes, sugars, nitrogenous compounds (such as spermidine alkaloids or muscarine), non-cannabinoid phenols, phenylpropanoids, steroids, fatty acids, and other compounds like stilbenes, lignans and phytosterols [4,5]. Among the most prevalent cannabinoid class, trans-Δ^9^-tetrahydrocannabinol (Δ^9^-THC) and cannabidiol (CBD) are well recognised for their psychotropic and therapeutic properties [6,7]. The others include cannabigerol (CBG), cannabichromene (CBC), (−)-Δ^8^-trans-tetrahydrocannabinols (Δ^8^-THC), cannabicyclol (CBLs), cannabielsoin (CBE), cannabinol (CBN), cannabinodiol (CBND), cannabitriol (CBT), and the miscellaneous cannabinoids [5,8]. Cannabinoids biosynthesis is primarily localised on the capitate-stalked trichomes mainly found on the flower and sugar leaf surface of the pistillate plant [9]. Disk-like structures formed on the trichome head synthesizes and secretes the cannabinoids which are then accumulated in the fibrillar matrix, the subcuticular wall, and the cuticle [9,10]. Shortly after the recoveries of the cannabinoids, the cannabinoid receptors (CB1 and CB2) were identified and many endogenous ligands for the cannabinoid receptors were also recognised [11]. They are collectively known as the “endocannabinoids” which are produced under the stimulation and released immediately from neurons [12]. Endocannabinoids are thought to play a role in wide range of physiological processes including pain reduction, mobility, learning recognition and rewarding. Recently, the pathological alteration of cannabinoid signaling has been elucidated not only in psychiatric complications but also many non-communicable diseases (NCDs) such as stroke; cancer; reproductive, cardiovascular, gastrointestinal disorders and neurodegenerative conditions such as Parkinson’s and Alzheimer’s diseases and multiple sclerosis [11,13]. Consequently, the endocannabinoid signaling pathway mainly from the major anandamide (AEA) and 2-arachidonoylglycerol (2-AG) are targeted as a cornucopia of therapeutic potential. Although Δ^9^-THC and CBD are well-studied phytocannabinoids from the cannabis species, new chemotypic structures have been characterised either from the new bred varieties of the cannabis or from other plant origins [14]. While many countries permit legal access to and use of botanical cannabis and its phytocannabinoid concentrates for medicinal purposes, the restrictions still prevail in some regions [15,16,17]. In Thailand, for example under the 2021 state law, some parts (mainly inflorescence) with high cannabinoid content are still prohibited for distribution, consumption and possession [15]. Consequently, in the light of the cannabinomics, the new inventory of phytocannabinoids along with their pathological activities should be updated particularly from non-cannabis sources [18]. Furthermore, the booming of the functional ingredients industries has prompted the new market for cannabinoids supplemented food and beverage products. This review attempts to compile knowledge on cannabinoid metabolites from non-cannabis plants that can be utilised as a baseline for cannabinoid research and development.

## 2. Endocannabinoid System

**Figure 1:**
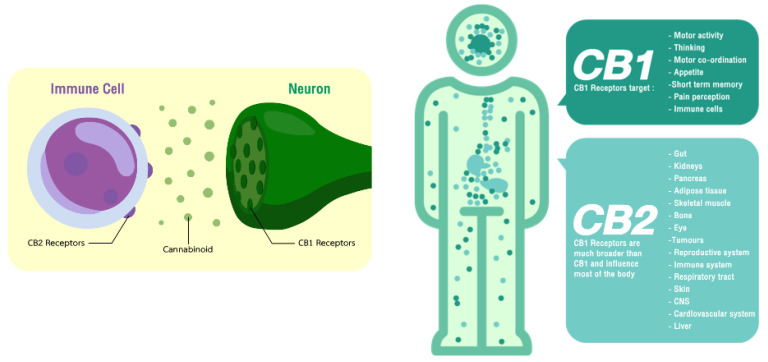
Localisation of the cannabinoid receptors and their therapeutic targets.

Human brain receptors for cannabinoids are present in all vertebrate and invertebrate animals with the exception of Protozoa and insects [19]. These receptors thereafter called CB1 and CB2 are the members of the G-protein coupled receptor (GPCR) family that mediate the biological effects with the endocannabinoids [20]. CB1 is the most common subtype in the central nervous system (CNS) and is also expressed throughout the human body [21]. It has attracted considerable interest as a potential therapy for a range of conditions, including neuropsychological problems and neurodegenerative diseases. CB2 is mostly found in the immune system and to a lesser extent in the central nervous system (Figure 1). CB2 has become the therapeutic target for immunomodulation, neuropathic and inflammatory pain, neuroinflammation, and neurodegenerative disorders. AEA was the first endogenous ligand found in pig brain after the discovery of these receptors [22]. Later, another endogenous cannabis molecule known as 2AG was identified [23,24]. These two forms of endogenous cannabinoid-like structures were derivatives of arachidonic acid, while 2AG is the most prevalent endocannabinoid in the human brain. The reference of the endocannabinoid system (ECs) is the combinations of CB receptors, their ligands, structure–affinity and all the enzymes and proteins that regulate cannabinomimetic properties [25,26]. The biosynthesis of these endocannabinoids occurs upon postsynaptic neuronal depolarisation and calcium influx. *N*-acylphosphatidylethanolamine-hydrolyzing phospholipase D (NAPE-PLD) and diacylglycerol (DAG) lipase are then activated by the calcium ion, forming AEA and 2-AG, respectively. The levels these endocannabinoid constitute varies depending on the tissue in which they are located [19]. The specific enzymes involved in altering this endocannabinoid tone are fatty acid amide hydrolase (FAAH) and monoacyl-glycerol lipase (MAGL), which are AEA and 2-AG specific Endocannabinoid, and they have been found in a variety of biological sources and have been shown to bind the receptors, eliciting signal transduction pathways and have a wide range of effects in peripheral tissues, illustrating numerous pharmacological effects. They also regulate numerous biological functions in humans, including memory, mood, reward systems and energy metabolism [8]. Despite their medicinal potential, the psychotropic properties of the cannabinoids have mainly limited usage in clinical practice [25].

## 3. Mass Spectrometry-Based for Discovery of Phytocannabinoids

**Figure 2:**
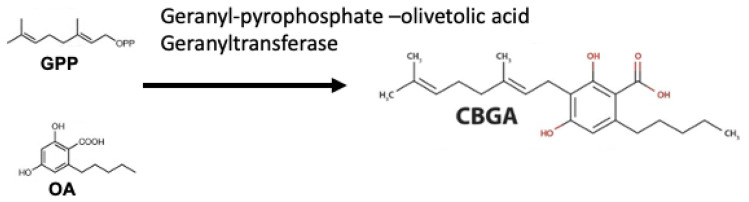
Cannabinoid biosynthesis begins with the combination of geranyl pyrophosphate (GPP) and olivetolic acid (OA) to form cannabigerolic acid (CBGA). CBGA serves as the substrate for phytocannabinoid synthesis.

Metabolomic research employs two complementary methods. Metabolic profiling examines a group of metabolites from a metabolic pathway or a class of compounds. The second method is metabolic fingerprinting. Initially, this approach compares patterns or “fingerprints” of metabolites that change in response to disease, toxin exposure, environmental or genetic alterations [27,28]. Mass spectrometry-based metabolics has proven to be extremely powerful in screening samples for a variety of signature patterns or clusters for metabolomic study. Among other approaches like GC-MS and LC-MS, NMR is accustomed to less tractable compounds such as sugars, amines, volatile ketones and relatively non-reactive compounds [29]. Originally, phytocannabinoids were meroterpenoids with a resorcinyl core typically decorated with a para-positioned isoprenyl, alkyl, or aralkyl side chain uniquely biosynthesised by *C. sativa* L. plants [8,30]. They are capable of directly interacting within the ECs. The phytocannabinoid structural motif results from the convergence of the mevalonate and the polyketide pathways (Figure 2). In phytocannabinoid profiling, GC and LC-based approaches have attained equivalent accuracy, selectivity, linearity, sensitivity, and precision, and are used in both routine and exploratory analysis of cannabis and cannabis-based products [31]. Mass spectrometry is used for confirmation and identification of the compounds due to the increased selectivity but also for quantitative analysis, due to the better sensitivity over other detectors [32]. The metabolic fingerprint of the examined sample is represented by *m*/*z* values, retention times, and intensities, which are exported for sample categorization utilising multivariate data analysis. Consequently, approximately 120 known phytocannabinoids which make up to about 24% of the total natural products of *C. sativa* have been found [33]. The chemical structure class within the Cannabaceae is diversely based upon their derivation from a common C21 precursor and the variation of the polyketide starter and prenyl oligomerisation as mentioned in Thomas and ElSohly [34]. Phytocannabinoids derived from aliphatic ketide starters are only found in *C. sativa* and have a limited natural distribution, known as the alkyl types, whereas, the analogues with an aralkyl-type substituent produced from an aromatic ketide starter have a significantly larger distribution, embracing not only plants but also liverworts and fungi [14]. The chromophore groups or fluorescent moieties such as the benzene structures in Δ^9-^THC, AEA and 2AG makes the direct detection under UV and GC-MS possible, however, the derivatisation step may also be required [35]. This may, however, interfere with the genuine cannabinoid content and also consume more time and increase cost. Alternatively, LC coupled with MS in selected ion monitoring (SIM) mode and MS2 in multi reaction monitoring (MRM) mode are the most commonly used detectors along with ultra-high performance liquid chromatography-tandem mass spectrometry (UPLC-MS/MS) and LC quadrupole-time of flight MS [36,37]. However, the matrix complexion can significantly affect the ion sources such as electrospray ionization (ESI) and atmospheric pressure chemical ionization (APCI). Therefore, it is critical to have a pretreatment capable of efficiently removing interfering compounds that cause ion suppression/enhancement [38].

Additionally, the advancement of metabolomic tools has prompted the new discovery of various natural cannabinoids from non-cannabis plants, in addition to the terpenophenolic constituents of the Δ^9^-THC and several of its naturally occurring derivatives [26]. Many of these chemicals are referred to as prenylated bibenzyls in the literature, a designation that masks their link to their more well-known cannabis equivalents. As a result, the term phytocannabinoid is inherently ambiguous in terms of structure. Presently, eleven distinctive classes of phytocannabinoids have been identified (Table 1) [39,40]. The Δ^9^-THC class represents the largest proportion, followed by the cannabigerol. These phytocannabinoid subclasses are proportionally varied depending on the growing conditions, geographical location, methods of extraction, and the varieties which influence the pharmacological effects the phytocannabinoid mix or entourage with non-cannabinoid content of the plant [4,33,40].

The cytoplasm of gland cells, the plastids, and the extracellular storage cavity are locations where phytocannabinoid production takes place. Hexanoic acid (C6) is produced in the cytosol by the oxidative cleavage of fatty acids (C18) such as palmitic acid; it is then synthesised into olivetolic acid (OA) via enzyme desaturase, lipoxygenase (LOX), and hydroperoxide lyases. In the plastid, the prenylation of phenolic moiety (the polyketide derivatives, 5-pentenyl resorcinolic acid, and OA) with the terpenoid geranyl pyrophosphate (GPP) happens as a result of the methylerythritol-4-phosphate (MEP) pathway [41]. The reaction of geranyl pyrophosphate (a terpenoid molecule) with either a C10 polyketide for the propyl (C3 side chain) or a C12 polyketide for the pentyl (C5 side chain) cannabinoid series produces either cannabigerolic or cannabidivaric acid [40]. Enzymatic conversion of these compounds produces a wide variety of C21 terpenophenolics including Δ^9^-THC, CBG, CBC, CBL, CBD, CBND, CBN and their C19 homologs, namely, Δ^9^-tetrahydrocannabivarin (Δ^9^-THCV), cannabivarin (CBV), and cannabidivarin (CBDV) [34].

## 4. Phytocannabinoids from Non-Cannabis Plant Origins

As said previously, the purpose of metabolomics is to analyse a wide range of metabolites in biological samples in a qualitative and quantitative manner [27]. High resolution mass spectrometry (HR-MS) in couplings with LC has become the most popular way for characterisation of the phytocannabinoids, as well as other metabolites in cannabis plants and other biological samples [32,42]. While NMR is sensitive, suited for quantitative studies, and elucidates structure along with and stereochemistry of an unknown compound, it is not selective and is also expensive for routine analysis [43]. Therefore, it has been extensively used for discovery of novel phytochemical structures. Besides, *C. sativa* rhododendrons produce exclusively alkyl phytocannabinoids, whereas aralkyl phytocannabinoids are common in the leguminous species and some others [44].

## 5. Non-Cannabis Sources

### 5.1. Rhododendron Species (Ericaceae Family)

Rhododendron plants belonging to the woody family of Ericaceae are the largest genus comprising of more than 1100 species worldwide [45,46]. They are also known as either medicinal or ornamental plants for attractive blight colour flowers and come in a variety of forms. The plant is also used in traditional medicines, which have been used for many years for the treatment of inflammation, skin or gastrointestinal tract disorders, particularly in Asia such as the Chinese and Ayurvedic medicine due to different secondary metabolites presented [47]. Bioactive diterpenoids, triterpenoids and polyphenolics have been previously elucidated from this genus [41,48]. The presence of the specific mono-, di-, or sesquiterpenoids in crude extract makes rhododendron plants a good candidate for antibacterial agents [49]. It is used as an expectorant and for chronic bronchitis in traditional medicine together with its anti-inflammatory properties for treating rheumatoid arthritis [48,50]. Grayanotoxin, which is the toxic diterpene mostly found in the flowers of several species, has been of interest lately and trace amounts have been detected in raw honey [51].

Rhododendron meroterpenoids from the twigs and leaves of *Rhododendron anthopogonoides* Maxim. have been characterised for the first time by comparison of their ^1^H-NMR spectral data which were structurally confirmed as cannabichromene (CBC), cannabicyclol (CBL), and cannabicitran (CBT) (Table 2). The fractions had also illustrated inhibitory effects on histamine release [50]. Twenty meroterpenoids from these plant parts were identified, including eight pairs of meroterpenoid enantiomers recovered by chiral-phase HPLC and four achiral meroterpenoids [48]. In this work, the first rhododendron meroterpenoids, with a hexahydroxanthene motif and a diterpene unit, had been found. The anthoponoids E, G and H had also suppressed the LPS-induced inflammatory responses in RAW 264.7 macrophages. In addition, daurichromenic acid (DCA), the meroterpenoid consisting of orsellinic acid and sesquiterpene moieties, that analogued to the cannabinoid structure was also found [52].

*R. dauricum* L., widely spread throughout northeastern Asia, also produces unique secondary metabolites including DCAs (Table 2) [52]. The MeOH extract of the leaves and twigs had illustrated significant anti-HIV activity [53]. In addition, the evidence showed that DCA possessed antibacterial and antifungal activities, therefore it could be the product of a plant defense mechanism [54]. DCA synthase has been also isolated from young leaves of *R. dauricum*, and it is important to highlight that the catalytic characteristics of this DCA synthase are remarkably comparable to those of cannabinoid synthases found in the cannabis plants [55]. Six chromene and chromene meroterpenoids including rubiginosins along with anthopogochromenes were isolated from the flowers of *R. rubiginosum* Franch. var. rubiginosum. These meroterpenoids were categorised as in the (CBC)-type and cannabicyclol (CBL)-type based on the isoprenyl moiety topical arrangement [56]. The fractions had illustrated low cytotoxicity against four human tumor cell lines. *R. capitatum* Maxim. is distributed and found particularly in alpine grasslands, meadows, and humid grasslands at an altitude above 2500 m [57]. Its aerial part had been used to isolate chromene meroterpenoids known as (+)−/(−)-rhodonoids and capitachromenic acids [58,59,60]. The later had also shown α-glucosidase and protein-tyrosine phosphatase 1B (PTP1B) inhibitory activity [59]. The inhibition of PTP1B consequently prolongs the efficiency of insulins in glucose homeostasis.

### 5.2. Other Angiosperm Species

The South African indigenous everlasting plant, *Helichrysum umbraculigerum* Less (Asteraceae family) and many species of the genus Helichrysum are widely used as traditional medicine. Besides many secondary metabolites found, the plant also consists of glandular trichomes in almost all vegetative epigeal parts, which are the source of essential oils [61,62]. Some African Helichrysum species are also used for ritual fumigations and recreational narcotics [44]. *H. umbraculigerum* has been found to contain phytocannabinoids in both the alkyl and aralkyl forms and cannabigerol has been reported to be the most abundant (Table 3). Cannabigerol is the precursor of all members of the alkylcannabinoids. Nonetheless Pollastro et al. [44] argued that the presence of bibenzyl resorcinoid, the lack of Δ^9^-THC or related alkyl-type cannabinoids make inhaling its vapour unlikely to produce narcotic effects. In a different study, CBG and CBGA (~0.2% of the aerial parts) as well as ‘abnormal’ CBGA are identified in *H. umbraculigerum* (Table 3). It is also unclear that the various oxidised forms of cannabigerol are natural products or rather isolation artifacts. This abnormal form was due to geranylation of olilvetol that produced CBG and its positional isomer [14]. Two amorfrutin-type phytocannabinoids were also elucidated, one with 2-methylbutanoyl esterification and another one featuring cyclisation of the prenyl unit to a chromane-type.

The edible root of licorice, *Glycyrrhiza foetida* Desf. (Fabaceae family) and the fruit of bastard indigobush, *Amorpha fruticosa* L. (Fabaceae family), are significant sources of amorfrutins, the active ingredients with a cannabinoid backbone as shown in Table 3 [44,63]. The cannabinoid structures carry an aralkyl side chain, providing facile access to resorcinol precursors of cannabinoids, and are thus categorised as prenylated bibenzyls [8,64]. These amorfrutins are clinically proven to have antidiabetic and lipid-lowering potentials [63,65]. In animal studies, the natural forms of amorfrutin A and B increased insulin production by targeting the peroxisome proliferator-activated receptor gamma (PPARγ), regulating fat and glucose metabolism, and further down streaming the inflammation [64]. The terpenolic component and active system for cannabinoid biosynthesis along with the m/z value of the cannabidiol-like structure had also been detected in the flax plant, *Linum usitatissimum* Linn. (Linaceae family) [66,67,68]. The isolated fraction also regulated inflammatory related genes including MCP-1, IL6 and SOCS-1 in animal trials, which confirmed the cell immunological response of the CBD-like structure [68].

### 5.3. Liverworts (Radulaceae Family)

Bryophytes are an early-diverged lineage of non-vascular, spore-forming plants that are distinctive and comprise more than 20,000 species [67]. They are also known as the first plants found on earth during the Cambrian period (~ 543–490 million years ago) [69]. They are placed in the plant kingdom in between algae and pteridophytes and are categorised into three classes; including mosses (Bryophyta), liverworts (Marchantiophyta), and hornworts (Anthocerotophyta) [70]. Liverworts are the most abundant phylum, comprising as many as 9000 species with high diversity in their ecology, morphology and genetic variation, and consequently are used the studies of the evolutionary origins of biodiversity and plant chemistry [67,69]. The most commonly recognised Radula species are found in all ecosystems such as trees, rocks, and soils throughout the world, from Antarctica’s coastal area to the northern hemisphere and from Australian semi-arid regions to the Amazon rainforest [71].

**Figure 3:**
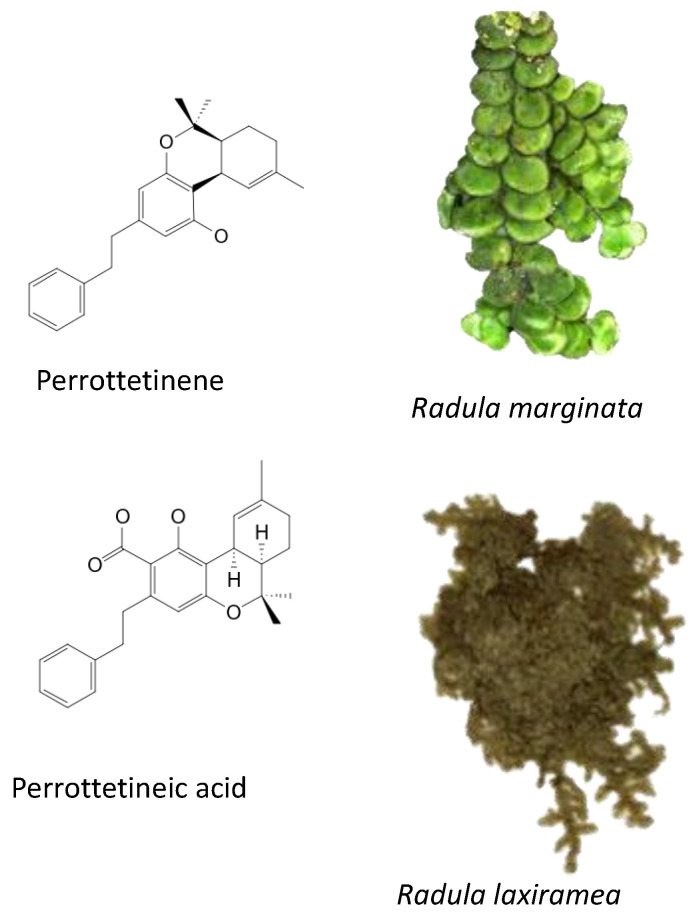
Phytocannabinoids in *Radula marginata* and *R. Laxiramea*.

Produced in the sac-like structures, known as oil bodies, liverworts are reported to be a rich source of secondary metabolites, including the nitrogen-containing alkaloids (especially, indole alkaloids), terpenoids, flavonoids and bibenzyl cannabinoids, especially in *Radula marginata* Hook.f. & Taylor [72]. These structures are localised in the center of the cell, which is a prominent and highly distinctive organelle unique to the liverworts [69,70]. Perrottetinene (PET) and perrottetineic acid, which are structurally analogous to ∆^9^-THC were isolated from some *Radula* spp. (Figure 3) [70,73]. These compounds have opposite stereochemical configuration (a cis configuration) in the cyclohexene ring compared with Δ^9^-trans-THC [71]. Most notably, (-)-cis-PET and its (-)- trans diastereoisomers demonstrated potential agonist toward CB1 and CB2 receptors. These compounds reduced basal prostaglandin levels (PGD2 and E2) in the brain in a CB1 receptor dependent manner, potentially imitating the action of 2-AG [70,74]. By using a de novo approach, the transcriptome of *R. marginata* was developed [67]. The upstream genes of the central precursor of cannabinoid biosynthesis, cannabigerolic acid (CBGA) biosynthesis including stilbene acid (SA) and geranyl diphosphate (GPP) intermediates were validated. Additionally, a homolog structure stilbene synthase (STS) that was a homolog of olivetolic acid had been characterised. Thus, (-)-cis-PET is a psychoactive cannabinoid from bryophytes, indicating the convergent development of bioactive cannabinoids in plants [71]. Beside PET, a chromene -like structure had been also found in *R. laxiramea* Steph. (Figure 3) [75].

## 6. Legal Consumption

Cannabis is known as the most frequently used illicit drug worldwide [76]. Federal and state governments have made their own regulations to protect public health and safety, including limiting access to minors, reducing the harms associated with illegal sales, and discouraging drunk driving. The Δ^9^-THC remains the principal psychoactive ingredient, while trace amounts (i.e., <0.2%) and the CBD are legal for medical uses in many countries such as several US states and other jurisdictions in Europe and South and Central America (Portugal, Spain, Belgium, Portugal, Argentina, Colombia, Jamaica) and Asia (Thailand) [15,77]. Non-medical use has only been legalized at a federal level in two countries, Uruguay and Canada, while decriminalized personal use had been legislated in the Netherlands [78,79]. Growing cannabis plants that contain <0.2% Δ^9^-THC (referred to as hemp) is legal, and the sale of hemp-based products, if they contain any detectable amount, are also allowed in some countries like Thailand [15,78]. The legalization framework focuses on the appropriate regulations concerning the legal age of possession, retail structures, the home growing of cannabis plants, permitted places for consumption, and cannabis-specific impaired driving laws [76], while less attention has been paid particularly to the psychoactive activity of the phytocannabinoids from non-cannabis organisms [71,80]. Chicca et al. [75] reported that cis-PET is a moderately potent but efficacious psychoactive cannabinoid that has been identified. Dried *R. marginata* collected in the wild is currently sold on the internet as a legal product, making reference to cis-PET being structurally similar to THC. A legal cannabis-like high can be obtained by consuming *R. marginata* and preparations, according to worldwide anecdotal reports [44,71,81]. Consequently, Food and Drug Administration (FDA)–approved medications have strict guidelines as to the variability in the content of their active moieties and their biochemical attributions with any of its main constituents [77]. We suggest that besides the advancement in cannabinomics and their inventory, the pharmacological studies of the phytocannabinoid from non-cannabis sources should be undertaken.

## 7. Conclusions

The advancement of metabolomics has prompted the analysis of novel phytochemicals from many biological samples in a qualitative and quantitative manner. Chromatography coupled with high resolution mass spectrometry with the variation of detectors has become the most common method for characterizing phytocannabinoids and other metabolites in non-cannabis plants. The rhododendrons produce exclusively alkyl phytocannabinoids, whereas aralkyl phytocannabinoids have been found in other angiosperm species along with the bryophytes with pharmacological properties. While the finding of these active ingredients may encourage the legal used of the phytocannabinoids for recreational purposes, we highlight that the further research on psychoactive activity of the phytocannabinoids from non-cannabis organisms should be given top priority.

## Figures and Tables

**Table 1:** Phytocannabinoid classes.

Compound Classes	Chemical Structure	Number of Compounds *	Therapeutic Activity
Δ^9^-trans-tetrahydrocannabinol	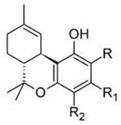	**9 subclasses** Δ^9^ tetrahydrocannabinol and its derivatives; R_1_ = C_5_H_11_, R_2_ = H Δ^9^ tetrahydrocannabinolic acid B; R_1_ = C_5_H_11_, R_2_ = COOH Δ^9^ tetrahydrocannabinol-C4 and its derivatives; R_1_ = C_4_H_9_, R_2_ = H or COOH Δ^9^ tetrahydrocannabivarin and its derivatives; R_1_ = C_3_H_7_, C_2_ = H Δ^9^ tetrahydrocannabiorcol and its derivatives; R_1_ = CH_3_, R_2_ = H or COOH	Euphoriant, analgesic, anti-inflammatory, antioxidant, antiemetic
Δ^8^-trans-tetrahydrocannabinol	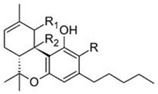	**2 subclasses** Δ^8^ tetrahydrocannabinol and its derivatives; R_1_ = COOH, R_2_ = C_5_H_11_	Euphoriant, analgesic, anti-inflammatory, antioxidant, antiemetic
Cannabidiol	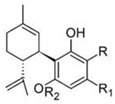	**7 subclasses** Cannabidiol and its derivatives; R_1_ = C_5_H_11_; R_2_ = H Cannabidivarin and its derivatives; R_1_ = C_3_H_7_, R_2_ = H	Antibiotic, antixiolytic, antiphychotic, analgesic, antioxidant, antispasmodic, anti-inflammatory
Cannabigerol	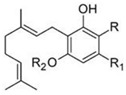	**6 subclasses** Cannabigerol and its derivatives; R_1_ = C_5_H_11_, R_2_ = H Cannabigerol monomethyl ether and its derivatives; R_1_ = C_5_H_11_, R_2_ = CH_3_ Cannabigerovarin forms; R_1_ = C_3_H_7_, R_2_ = H	Antibiotic, antifungal, anti-inflammatory, analgesic
Cannabichromene	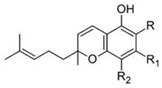	**4 subclasses** Cannabichromeme and its derivatives; R_1_ = C_5_H_11_, R_2_ = CH_3_ Cannabichromevarin and its derivatives; R_1_ = C_3_H_7_, R_2_ = CH_3_	Antibiotic, antifungal, anti-inflammatory, analgesic
Cannabinol	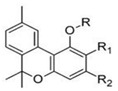	**9 subclasses** Cannabinol and its derivatives; R = H, R_1_ = H or COOH, R_2_ = C_5_H_11_ Cannabivarin; R = H, R_1_ = H, R_2_ = C_3_H_7_ Cannabiorcol; R = H, R_1_ = H, R_2_ = C_2_H_5_ Cannabinodiol; R = C_5_H_11_ Cannabinodivarin; R = C_3_H_7_	Sedative, antibiotic anticonvulsant, anti-inflammatory
Cannabinodiol	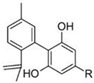		
Cannabicyclol	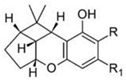	**3 subclasses** Cannabicyclol and derivatives; R_1_ = C_5_H_11_ Cannabicyclovarin; R_1_ = C_3_H_7_	
Cannabielsoin	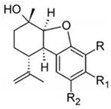	**3 subclasses** Cannabielsoin and its derivatives; R_1_ = C_5_H_11_, R_2_ = H or COOH	
Cannabitriol	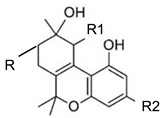	**5 subclasses** Cannabitriol; R = H, R_1_ = OH, R_2_ = C_5_H_11_ 10-ethoxy-9-hydroxy Δ^6^a- tetrahydrocannabinol; R = H, R_1_ = OC_2_H_5_, R_2_ = C_5_H_11_ 8,9-hydroxy Δ^6a^- tetrahydrocannabinol; R = OH, R_1_ = H, R_2_ = C_5_H_11_ Cannabitroolvarin; R=H, R_1_ = OH, R_2_ = C_3_H_7_ Ethoxy-cannabitriolvarin; R = H, R_1_ = OC_2_H_5_, R_2_ = C_3_H_7_	
Unclassified types	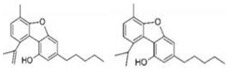	- For examples; de-hydrocannabifuran DCBF-C5, cannabi-furan CBF-C5-	

* R = COOH (acid form); R = H (neutral form) except those from the cannabinol, cannabinodiol and cannabitriol classes.

**Table 2:** Phytocannabinoid characterisation from different *Rhododendron* spp.

*Rhododendron* spp.	Plant Part Used for Extraction	Mass Spectrometry-Based Metabolomics	Novel or Specific PhytocannaBinoid Identified
*R.* *anthopogonoides* 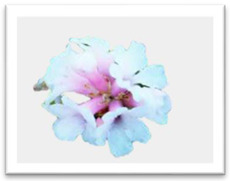	twigs and leaves [48,49]	HR-FAB-MS with UV, IR and ^1^H-NMR spectrums [50] HR-ESI-MS couple with 1D NMR, and HSQC spectra [48]	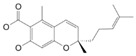 Anthopogochromenic acid 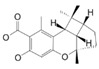 Chromene derivative (anthopogocyclolic acid)
*R. dauricum* L. 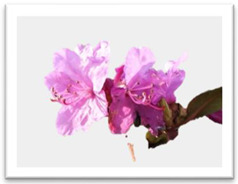	Leaf	molecular formula was established by HR-FAB-MS coupling with ^1^H-NMR and ^13^C spectra [53].	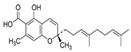 Daurichromenic acid 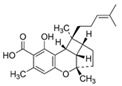 Rhododaurichromanic acid A 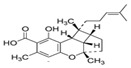 Rhododaurichromanic acid B
*R. rubiginosum* 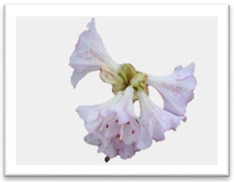	Flower	molecular formula was established by HR-ESI-MS, couple with IR, UV 1D and 2D NMR spectras. The planer structure was estab-lished by its ^1^H-^1^H COSY and HMBC spectra [56].	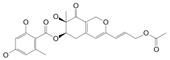 Rubiginosin and its derivatives
*R. capitatum* 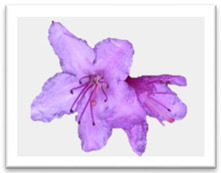	Aerial part	HR-MS with solid phase extraction coupled with 1H and ^13^C NMR [59].	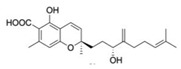 Capitachromenic acid A and its derivatives

**Table 3:** Phytocannabinoid structures from different angiosperm species.

Everlasting, *Helichrysum umbraculigerum* 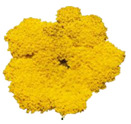	Licorice, *Glycyrrhiza foetida* 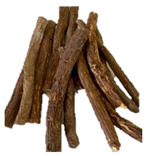	Bastard indigobush, *Amorpha fruticose* 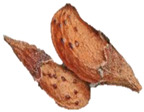	Flax fibre, *Linum usitatissimum* 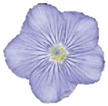
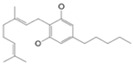 Cannabigerol and its derivatives 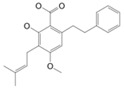 Amorfrutin A and its derivatives	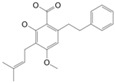 Amorfrutin A 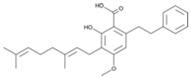 Amorfrutin B		Non-characterised compound
